# From compound to target: chemical proteomics and *in silico* screening identify Hsp90 and CDPK2 as putative targets in *Plasmodium falciparum*

**DOI:** 10.1186/1475-2875-11-S1-P60

**Published:** 2012-10-15

**Authors:** Leonardo Lauciello, Shaheen Ahmed, Bianca Derrer, Barbara Kappes, Tai Wang, Werner Seebacher, Leonardo Scapozza, Remo Perozzo

**Affiliations:** 1 Pharmaceutical Biochemistry Group, School of Pharmaceutical Sciences, University of Geneva & Lausanne, Quai Ernest-Ansermet 30, 1211 Geneva, Switzerland; 2 Institute of Medical Biotechnology, University FAU Erlangen-Nüg, Paul-Gordan-Str. 3, 91052 Erlangen, Germany; 3 Department of Cell Biology, University of Geneva, Quai Ernest-Ansermet 30, 1211 Geneva, Switzerland; 4 Institute of Pharmaceutical Sciences, Pharmaceutical Chemistry, Karl-Franzens-University Universitätsplatz 1, 8010 Graz, Austria

## Background

For several years now, *Plasmodium falciparum* is developing resistance to drugs in use. There is hence an urgent need for new treatments as well as new targets. By identifying the targets of novel or known active molecules with unknown mechanisms of action, it is possible to guide the development of new chemical entities towards their clinical application. This project aims at finding the putative target(s) of CP1, a new molecule in the development phase, and of triclosan, a well-known antibacterial and fungicide, by means of chemical proteomics and ligand based inverse virtual screening.

## Materials and methods

The parasite lysate was incubated with the affinity matrix and retained proteins were analyzed by LC-MS/MS. Yeasts complemented with plasmodial Hsp90 were grown in minimal media. Viability was calculated by comparison with untreated strains. Binding of CP1 derivatives to purified N-terminal PfHsp90 was evaluated with Differential Scanning Fluorimetry. For the identification of potential triclosan binders, molecules were evaluated *in silico* using molecular docking program GOLD. Inhibition of PfCDPK2 and mechanism of action of triclosan were evaluated with a radiometric assay using myelin basic protein (MBP) as substrate.

## Results

For CP1, chemical proteomics identified heat shock protein 90 (Hsp90) as a putative binder. Subsequent assays confirmed that the viability of yeast cells where the wild-type Hsp90 has been substituted with the plasmodial one was strongly reduced in presence of CP1. Moreover, CP2 was proven to bind the N-terminal domain of PfHsp90. R triclosan, the *in silico* inverse screening proposed the calcium-dependent protein kinase 2 (PfCDPK2) as its potential binding partner. Enzymatic assays confirmed inhibition of PfCDPK2 with an IC_50_ of 48 µM. Furthermore, the mechanism of action was determined to be non-competitive towards ATP.

## Conclusion

This study shows that both chemical proteomics and *in silico* approaches are valuable tools for the identification of potential targets or binders of active molecules. The results obtained so far for PfHsp90 point definitely towards an interaction with the protein, although a direct proof of inhibition is still needed. On the other side, the confirmation of the inhibition of PfCDPK2 by triclosan opens new perspectives in the use of this molecule and derivatives thereof against *Plasmodium falciparum.* In both cases, such results represent a starting point towards the optimization of the molecules and the development of new therapeutics against malaria.

